# Exploring Microbial Diversity and Yeast Typing in Traditional Sourdoughs from Villaurbana (Sardinia, Italy) Using an Integrated Approach

**DOI:** 10.3390/foods15132307

**Published:** 2026-06-29

**Authors:** Roberta Coronas, Anna Maria Laura Sanna, Roberto Cabizza, Anna Reale, Angela Bianco, Cécile Grondin, Jean Luc Legras, Giacomo Zara, Marilena Budroni

**Affiliations:** 1 Department of Agricultural Sciences, University of Sassari, 07100 Sassari, Italy; 2 Institute of Food Sciences, National Research Council (CNR-ISA), Via Roma 64, 83100 Avellino, Italy; 3 Institut National de Recherche pour l’Agriculture, l’Alimentation et l’Environnement (INRAE), Unité Mixte de Recherche—Sciences pour l’Œnologie (UMR SPO), 34060 Montpellier, France

**Keywords:** citizen science, co-designing intervention, type I sourdough, sourdough mycobiota, microsatellite yeast typing

## Abstract

Sourdoughs are complex microbial ecosystems fundamental to traditional breadmaking. Despite extensive research, variability among traditional sourdough ecosystems remains a key area of investigation. In this context, 13 type I sourdough starters from Villaurbana (Sardinia, Italy) were characterised combining molecular and biochemical techniques. Bacterial and fungal communities were identified by NGS-based amplicon sequencing, while lactic and acetic acid, and residual sugars were quantified. The bacterial population was dominated by the genus *Fructilactobacillus*, whereas the fungal community spanned multiple genera. To explore the mycobiota, 130 yeast isolates were identified sequencing D1–D2 domain and through MALDI-TOF mass spectrometry. Six yeast species were detected: *Saccharomyces cerevisiae* was the dominant species (58.46%), followed by non-*Saccharomyces* yeasts (41.54%), including *Torulaspora delbrueckii*, *Pichia fermentans*, *Wickerhamomyces anomalus*, *Maudiozyma humilis*, and *Monosporozyma unispora*. Strain-level typing via microsatellite analysis revealed high intraspecific diversity among *S. cerevisiae* and *T. delbrueckii isolates. S. cerevisiae* strains were distributed across distinct genetic lineages, with some clustering alongside industrial reference strains and others displaying unique evolutionary trajectories. *T. delbrueckii* strains formed two clonal groups substantially divergent from reference strains. This study supports the restitution of characterized yeast strains to the community as a resource for establishing a new microbial consortium representative of Villaurbana sourdough tradition.

## 1. Introduction

Fermented cereals are among the most widely consumed foods that have played an important role in traditional diets around the world since time immemorial [1]. Sourdough bread, obtained by the natural fermentation of flour, water and other ingredients, is one of the most traditional bakery products. It is distinguished by its appetizing, tasty and easily digestible qualities [2]. The use of sourdough in breadmaking significantly improves structural properties, such as volume, crumb softness and elasticity [3], as well as microbiological properties, including food safety and extended shelf life [4]. Moreover, sourdough enhances nutritional value, aroma and flavour diversity [5], and health-related effects such as digestibility and degradation of anti-nutritional factors [6].

The wide variety of traditional breads produced around the world is influenced by the diversity of raw materials used (such as water, flour and salt), fermentation practices, microbial biodiversity and baking techniques [7,8]. Sourdoughs are produced through spontaneous fermentation and consistently maintained through backslopping, a process shared by sourdough producers worldwide [9]. A persuasive example can be found in Sardinian sourdough culture, where Type I sourdough has played and continues to play a key role in the region’s culinary heritage, contributing to the preservation and innovation of traditional sourdough breads and strengthening the sustainability of grain-to-bread supply chains [10,11]. Sardinian sourdoughs are produced using semolina from durum wheat, particularly the Capelli variety, which thrives in the island’s arid Mediterranean climate and yields flour of the high quality for traditional breads and pasta [12]. The distinctiveness of Sardinian sourdough cultures further stems from the island’s microbial biodiversity [13]; notably, the use of brewer’s yeast remains a relatively recent introduction. In a regional context in which multiple, distinct crises (economic, ecological, energy-related, social, and demographic) occur simultaneously and interact in ways that amplify each other, these products can contribute to making the food system more sustainable and resilient. Strengthening collaboration and knowledge exchange among researchers, producers, local administrators, and citizens through citizen science approaches can support the sustainability of cereal fermentation practices and the preservation of local food traditions [14,15]. This is consistent with the recognition that citizen science methodology engaging local communities in data collection and knowledge sharing represents a valuable tool for documenting traditional fermentation practices [16,17,18] with many initiatives also striving to make their results openly accessible to foster broader scientific participation [19,20].

Prior studies on Italian sourdoughs have shown that ecosystems of sourdoughs are typically structured by a relatively limited core microbiota, with lactic acid bacteria such as *Fructilactobacillus sanfranciscensis*, *Lactiplantibacillus plantarum*, *Companilactobacillus paralimentarius*, and yeasts such as *Saccharomyces cerevisiae* and *Candida*/*Kazachstania* species frequently accompanying them [21,22]. Within this broader Italian framework, Sardinian sourdoughs have received comparatively less attention: a survey of 25 sourdough samples used to produce traditional breads identified 12 LAB species, with *Lactiplantibacillus pentosus* dominating many samples and *F. sanfranciscensis* occurring only in a limited number [23]; a separate Sardinian study targeted natural yeast characterisation and monitoring of inoculated strains by real-time PCR [24]. Overall, descriptive studies specifically focused on Sardinian sourdough microbiota remain scarce. Markedly, characterising yeasts in sourdough is essential to evaluate the microbial diversity that drives fermentation performance, flavour development, and the nutritional and safety properties of the final product [25]. Preserving and characterising yeast strains ensures the stability and reproducibility of the sourdough microbiota and supports the selection of functional starter cultures tailored to specific product traits. This provides the knowledge needed to optimise fermentation conditions and enhance bread volume, texture, aroma complexity, and consumer acceptance [26,27].

In this context, the MBDS-UNISSCC culture collection of the University of Sassari, part of the Sardinia region germplasm bank, has collected 109 sourdoughs from across Sardinia for the purpose of preserving and studying their microbiomes. One of MBDS-UNISSCC’s main goals is to make microbial cell factories accessible to stakeholders. The research infrastructure collaborated with the Municipality of Villaurbana (OR, Sardinia) to co-design a participatory study involving the rural community in the scientific exploration of its baking practices, which have involved the continuous maintenance and transmission of sourdough across generations.

This study aimed to characterise the microbial diversity of 13 Type I sourdoughs, analysed through an integrated molecular and biochemical approach. Bacterial and fungal communities were profiled by NGS-based amplicon sequencing, while yeast isolates were identified and typed using a multi-method strategy combining sequencing of the D1–D2 region of ribosomal DNA, MALDI-TOF mass spectrometry, inter-delta fingerprinting and microsatellite analysis for *Saccharomyces cerevisiae*, and *Torulaspora delbrueckii*. Organic acids and residual sugars were quantified to link microbial composition to fermentative activity. The overarching goal was to assess whether sourdoughs from a single municipality harbour sufficient yeast diversity to support the design of a locally representative microbial consortium for the preservation and valorisation of the Villaurbana sourdough tradition.

## 2. Materials and Methods

### 2.1. Sourdoughs

Thirteen traditional type I sourdoughs were collected in collaboration with “Comune di Villaurbana” (OR, Sardinia), as part of the project “TRIGU—Urban regeneration and enhancement of the historical, cultural, tangible and intangible heritage of the Municipality of Villaurbana”. Responding to a municipal call for proposals, citizens offered their sourdough starters, which were used for both household and commercial bakery production, to be studied and stored in the MBDS-UNISS Culture Collection for a period of thirty years. The samples were delivered in sterile containers, accompanied by a descriptive form detailing their main characteristics, as well as the practices of backslopping and usage. The flour used for backslopping was included with each sample to minimize alteration to the original microbial composition. All sourdough starters were transported under refrigerated conditions on the same day and named SD15, SD42, SD96, SD97, SD99, SD101, SD103, SD104, SD105, SD106, SD107, SD108, SD109, and stored at 4 °C until analysis (Table 1).

### 2.2. Physicochemical Parameters, Organic Acids and Sugar Profile

Prior to biochemical analysis, each sourdough was subjected to a single backslopping cycle in the laboratory using the flour provided by each household, under the fermentation temperature and time reported by each participant in the accompanying metadata form. Physicochemical and biochemical parameters were measured at the end of this propagation cycle. The pH of sourdough samples was determined through a pH-meter (SensION^TM^+ PH3, Hach Lange S.r.l, Lainate, MI, Italy) equipped with a food penetration probe. Total titratable acidity (TTA), expressed as the volume (mL) of 0.1 M NaOH required to achieve a pH of 8.3, was measured in 10 g of sourdough samples mixed with 90 mL of distilled water for 3 min in a bag mixer (400P; Interscience, St Nom, France).

The quantification of lactic acid (D-/L-lactic acid), acetic acid, glucose, sucrose, fructose, and maltose was performed using enzymatic assays with an I-Magic M9 automatic enzymatic analyzer (R-Biopharm AG, Darmstadt, Germany), following the manufacturer’s instructions. Specific enzymatic kits from the Enzytec™ Liquid line (R-Biopharm AG) were employed for each analyte, including kits for D-/L-lactic acid, acetic acid, and individual or combined sugar determination (glucose, sucrose, fructose, and maltose), based on UV-method detection at 340 nm via NADH formation. Prior to analysis, the instrument was calibrated using certified multi-analyte standards. Calibration for D-L-lactic acid was performed using Enzytec™ Liquid Multi-acid standards (low and high; art. no. E8460/E8465). Acetic acid calibration was carried out using the standard solution provided within the enzymatic kit (art. no. E8226). Calibration for sugars was performed using Enzytec™ Liquid Multi-sugar standards (low and high; art. no. E8440/E8445). Sample preparation was carried out according to Lefebvre et al. [28] as follows: 10 g of sourdough was suspended in 50 mL of distilled water at 50–60 °C and stirred with a magnetic stirrer in a heated water bath (50–60 °C) for 15 min. Subsequently, 5 mL of Carrez I reagent and 5 mL of Carrez II reagent were added sequentially. The solution was then adjusted to pH 7.0 with 0.1 N NaOH and brought to a final volume of 100 mL with water. The mixture was filtered through filter paper using a funnel. For acetic acid determination specifically, Carrez I reagent containing zinc sulfate instead of zinc acetate was used to avoid interference that would cause off-scale readings. The clarified samples were subsequently filtered through a 0.45 µm nylon membrane filter and directly analysed using iMagic M9. All measurements were performed in three technical replicates.

### 2.3. Total DNA Extraction, Library Preparation and Sequencing

Prior to subsampling, each sourdough sample was manually homogenized to ensure spatial representativeness of the aliquot. Eight grams of sourdough were mixed with 40 mL of sterile 50 mM potassium phosphate buffer (PBS) at pH 7.0 and homogenized. The samples were centrifuged (1300 rpm for 5 min) and the obtained pellets were subjected to total DNA extraction using the DNeasy PowerFood Microbial Kit (QIAGEN, Milan, Italy), according to the manufacturer’s instructions. For bacterial analysis, primers were designed to amplify the V3–V4 hypervariable regions of the 16S rRNA gene [29]. For fungal analysis, primers specific for the ITS1 region located between the 18S and 5.8S rRNA genes were used [30]. Sequencing library preparation and subsequent sequencing were conducted by BaseClear BV (Leiden, The Netherlands) utilizing the Illumina MiSeq platform with 300-cycle paired-end sequencing technology. Demultiplexing and generation of paired-end FASTQ files were performed by the sequencing provider. The demultiplexed FASTQ files were analysed using a DADA2-based workflow. Quality control of sequences and determination of amplicon sequence variants (ASVs) were accomplished in paired-end mode, applying a maximum expected error threshold of 5 and truncation at low-quality bases (truncQ = 4), paired-end merging with a minimum overlap of 12 bp, a minimum sequence length of 75 bp, and chimera removal.

For the bacterial (16S) dataset, the mean read count per library decreased from 61,562 ± 9232 reads in input to 59,739 ± 8945 reads retained after filtering (mean retention 97.0%). After denoising, paired-end merging, minimum-length filtering and chimera removal, the final non-rarefied non-chimeric ASV table comprised 128 ASVs and 1,537,842 reads, with sequencing depth ranging from 36,020 to 76,267 reads per library (median 60,557.5). After removal of chloroplast- and mitochondria-assigned ASVs, the taxonomically filtered 16S table used for downstream analyses comprised 65 ASVs and 1,486,883 reads, with sequencing depth ranging from 34,584 to 73,654 reads per library (median 59,177). For the fungal/yeast (ITS) dataset, the mean read count per library decreased from 64,844 ± 3172 to 64,167 ± 3138 reads after filtering (mean retention 99.0%). After denoising, paired-end merging and chimera removal, the final ASV table comprised 355 ASVs and 349,555 reads, with retained reads ranging from 601 to 39,158 reads per library (median 10,111.5).

Taxonomic assignment for bacterial sequences was performed using the RDP method with a bootstrap threshold of 40 against Greengenes2 (GG2) v202409 reference files [31]. Fungal/yeast ITS sequences were assigned against the UNITE fungi reference database (version 18.07.2023) [32] using a bootstrap threshold of 80. Taxonomic filtering criteria and the list of ASVs removed prior to downstream analyses are reported in the Supplementary Material (Tables S1–S6). For graphical representation of microbial community composition, ASV tables were first filtered by applying a relative abundance threshold of 2 × 10^−6^. The resulting ASV tables were then rarefied to the sequencing depth of the least abundant sample within each dataset. Relative abundances were calculated from the rarefied ASV tables and subsequently aggregated at genus level to generate the community composition plots.

### 2.4. Microbiological Characterization

The sourdoughs were subjected to culture-dependent microbiological analyses, following the Standard Operation Procedures (SOPs) for microbiome sampling [33]. Presumptive lactic acid bacteria (LAB) were enumerated through the plate count method after the incubation at 30 °C for 48 h using De Man-Rogosa-Sharpe agar (MRS) (VWR International Srl, Milan, Italy), supplemented with cycloheximide (0.1 g/L) (Sigma-Aldrich, © 2024 Merck, Darmstadt, Germany) under microaerophilic conditions generated using a GasPak system (BD GasPak™ EZ, Franklin Lakes, NJ, USA). Yeast cell density was estimated on Wallerstein Laboratory Nutrient Agar (WL) (VWR International Srl, Milan, Italy) supplemented with chloramphenicol (0.1 g/L) (Sigma-Aldrich, © 2024 Merck, Darmstadt, Germany) after incubation at 25 °C for 48 h. Microbial count was conducted in three technicals replicates.

### 2.5. Isolation and Identification of Yeast Isolates

Ten yeast isolates from each sourdough were streaked on WL medium consecutively to obtain pure cultures based on morphological differences, for a total of 130 isolates. It should be noted that this morphology-guided selection strategy may have underestimated the actual diversity within each sample, as strains sharing similar colony morphology on WL agar may not have been recovered. Consequently, culture-dependent diversity estimates should be interpreted as minimum estimates of the yeast diversity present. In this context, two identification methods were applied: sequence analysis of the LSU D1/D2 domain and MALDI-TOF MS identification. Pure colonies were inoculated into 3 mL of YPD broth (10 g/L yeast extract, 20 g/L peptone, 20 g/L dextrose) and incubated overnight at 25 °C while shaking at 200 rpm. Genomic DNA extraction was performed according to the method described by Lõoke et al. [34]. The PCR reaction for amplification of the D1/D2 region (50 μL total volume) contained 2 μL of genomic DNA template, 0.4 μL of 25 mM dNTPs, 2 μL each of primer NL1 (5′- GCATATCAATAAGCGGAGGAAAAG-3′) and NL4 (5′-GGTCCGTGTTTCAAGACGG-3′) at 5 pmol/μL, 5 μL of 10× buffer, 0.2 μL of Ex Taq polymerase (5 U/μL), and water to reach the final volume. The thermal cycling program consisted of an initial denaturation step at 94 °C for 4 min, followed by 30 cycles of denaturation at 94 °C for 30 s, annealing at 54 °C for 40 s, and extension at 72 °C for 1 min 30 s, with a final hold step at 4 °C. PCR products were verified by electrophoresis on 0.8% agarose gel in TAE buffer. Amplicons were then sequenced and analysed by BLAST algorithm (https://blast.ncbi.nlm.nih.gov; accessed on 1 December 2025) against the NCBI nucleotide database for species identification; a sequence identity threshold of ≥98% was applied for species-level identification.

Yeast samples were prepared for MALDI-TOF MS identification following the standard Bruker ethanol/formic acid extraction protocol [35]. Each dried spot was then overlaid with 1 μL of HCCA (α-cyano-4-hydroxycinnamic acid) matrix solution, which co-crystallized with the proteins during drying. This extraction method yielded a maximum number of ribosomal protein peaks in the mass spectra, enabling robust standardization and accurate microbial identification by MALDI Biotyper^®^ (Bruker Daltonics, Bremen, Germany) and compared to the Bruker database DBAL-13.0, that had been complemented with spectra from strains of the CIRM-Levures. Each isolate was analysed in four technical replicates (four spots per isolate); species-level identification was accepted when at least two out of four readings yielded a Biotyper log score ≥ 2.00. Any discrepancies between D1–D2 sequencing and MALDI-TOF MS identification were clarified by amplifying the DNA with sequencing of the ITS region.

### 2.6. Biotyping of Saccharomyces cerevisiae and Torulaspora delbrueckii Strains

*Saccharomyces cerevisiae* isolates were differentiated by inter-delta PCR fingerprinting, which amplifies regions between delta elements (long terminal repeats, LRTs) of Ty1 and Ty2 retrotransposons. The PCR reaction (50 μL total volume) contained 4 μL of genomic DNA template (25 ng), 0.4 μL of 2.5 mM dNTPs, 5 μL each of primers δ12 and δ21 [36] at 5 pmol/μL, 5 μL of 10× DreamTaq buffer, 0.4 μL of DreamTaq polymerase (5 U/μL), and 30.2 μL of water. The thermal cycling program consisted of initial denaturation step at 95 °C for 3 min, followed by 30 cycles of denaturation at 95 °C for 30 s, annealing at 45 °C for 40 s, extension at 72 °C for 1 min 30 s, with a final extension at 72 °C for 5 min and a hold at 4 °C. PCR products (15–20 μL) were analyzed by electrophoresis on 1.2% agarose gel in TAE buffer containing ethidium bromide at 110 V for 90 min. The resulting fingerprint patterns were analysed with Gelj V2.3 software [37] and a cluster dendrogram was obtained using the Dice coefficient with tolerance of 1.5.

*Saccharomyces cerevisiae* isolate set was further differentiated at the strain level using microsatellite typing, a more precise and discriminatory technique. Strain characterization was performed using two multiplex PCR reactions with fluorescently labelled primers. Primer stock solutions were prepared by combining forward and reverse primers at a concentration of 5 μM. Mix one contained primer for loci C8 (FAM), C11 (FAM), C5 (HEX), GAG (HEX), C3 (TAMRA), and SCAAT3 (TAMRA). Mix two included primers for loci C9 (TAMRA), YPL172 (FAM), C7 (FAM), C6 (HEX), C4 (TAMRA), SCAAT1 (HEX), and SCAAT5 (TAMRA). Each PCR reaction (12.5 μL per sample) contained 6.25 μL of Qiagen Multiplex PCR Mix (2×), 0.25 μL of primer mix (final concentration 0.1 μM), 1 μL of genomic DNA template, and 5 μL of water. The thermal cycling program consisted of initial denaturation at 95 °C for 15 min, followed by 34 cycles of 95 °C for 30 s, 57 °C for 2 min, and 72 °C for 1 min, with a final extension at 60 °C for 30 min. Amplicons were diluted 1:100 in distilled water, and 3 μL of diluted product was mixed with 17 μL of water containing 0.2 μL of GeneScan 400HD ROX size standard. Samples were denatured at 95 °C for 5 min, immediately cooled on ice, and analyzed by capillary electrophoresis on an ABI sequencer for microsatellite fragment sizing. The profiles obtained were subsequently compared with those of 43 reference strains of known origin (Table S7): 4 wine strains from Tuscany; 20 sourdough strains from Sicily; 8 sourdough strains from Sardinia, Lombardy and Sicily; 5 industrial bakery yeast strains from France, New Zealand and Morocco; 5 oak-associated strains from Pennsylvania and New Jersey (USA); and 1 commercial brewing strain.

The same procedure was applied to a set of 20 isolates of *Torulaspora delbrueckii*, using 7 loci described by Albertin et al. [38], and 3 new loci combined into two mixes. Mix one (TD1) contained primers for loci TD5A_GT (HEX), TD1A_CAA (HEX), TD1C_TTA (HEX), TD1B_GT (FAM), TD2A_GTT (FAM), TD6A_CAA/CAG (TAMRA), and TD4A_TA (TAMRA). Mix two (TD2) included primers for loci TD7A_TTAA (HEX), TD8B_TAA (FAM), and TD8C_CAA (HEX). The following primers have been designed specifically during this study: TD4A_TA (fw CTTCACTCACCAGATCAACA), rv GAAATGCTTATTTTTAAGGTGGGA), TD8B_TAA (fw: AGAACGAATSTCTTGGAAAATC, rv: GACTCTATGCATTTAACCCATC), TD8C_CAA (fw: CAAATGCTTATACCCCAATACC, rv: GCGACTCTTCTACCATTGATTA). The profiles were further compared against an additional set of reference profiles sourced from diverse ecological niches, including grapes, cheese, soil, and cider, for a total of 26 reference strains (Table S8).

### 2.7. Statistical Analysis

Physicochemical data (pH, total titratable acidity, organic acid concentrations, and residual sugar levels) were subjected to one-way analysis of variance (ANOVA). When significant differences were detected (*p* < 0.05), mean separation was performed using Tukey’s Honestly Significant Difference (HSD) post hoc test. All parametric analyses were carried out in R (version 4.3.1; R Core Team, 2023).

For microsatellite-based strain characterisation of *S. cerevisiae*, pairwise genetic distances between isolates were calculated using the Cavalli-Sforza and Edwards chord distance metric (Dc) implemented in a custom script [39]. A Neighbor-Joining (NJ) tree was constructed from the resulting distance matrix using “ape” package [40], which applies a star-decomposition algorithm robust to negative branch lengths. Bootstrap values are not reported for this tree, as standard bootstrap resampling is not applicable to microsatellite-based dendrograms with a limited locus set; robustness of major group differentiation is instead supported by the jackknife procedure described in Legras et al. [39] for the same locus panel, which confirmed the stability of major genetic group separation across iterative locus subsets. For *T. delbrueckii* isolates, pairwise genetic distances were calculated using the Dc chord distance metric, and a Neighbor-Joining tree was constructed following the same procedure. Bootstrap values are not reported for this tree, as several strains share identical multilocus genotypes whose clustering reflects arithmetic identity rather than phylogenetic inference and is therefore not subject to sampling uncertainty. Inter-delta fingerprint profiles were analysed using GelJ V2.3 software [37], and a cluster dendrogram was generated using the Dice similarity coefficient with a tolerance of 1.5%, applying the unweighted pair group method with arithmetic mean (UPGMA) algorithm.

## 3. Results and Discussion

### 3.1. Metabolic Dynamics in Villaurbana Sourdoughs: Biochemical Diversity and Acidification Profiles

Questionnaire data showed differences in starter origin (inherited and homemade sourdough), first fermentation conditions (at room temperature; from 3 to 48 h), backslopping frequency (from daily to weekly, or as-needed basis), storage methods (refrigeration and freezing), bread typologies (traditional breads such as “Civraxiu”, “Moddixia”, “Coccoi” and common bread). Villaurbana Type I sourdoughs show substantial consistency between the documented artisanal practices in Sardinia and the parameters characterizing this fermentation category globally [41]. Table 2 reports the pH and TTA and dough yield (DY) values measured in the 13 sourdoughs.

The Type I sourdough samples analysed show characteristics consistent with mature fermentation and well-managed backslopping procedures. pH measurements ranging from 4.06 to 4.64 and total titratable acidity (TTA) values ranging from 8.6 to 17.1 mL of 0.1 M NaOH, fall within the optimal ranges for stabilised, spontaneous sourdough starters [42], indicating an active and balanced microbial community (Table 2). The variation observed in TTA is significantly greater than the variability in pH (coefficient of variation: 22.6% vs. 3.9%, respectively), indicating that while pH values remain relatively stable across samples, the total acid content differs considerably. This suggests different compositions of organic acids among the samples, probably related to the different proportions of lactic and acetic acid produced by heterogeneous lactic bacterial communities [4]. Dough yield values (135–200), indicating medium-stiff dough hydration levels, aligned with standard management practices of Type I sourdough [43].

The variability observed across all parameters suggests that multiple management approaches can maintain viable sourdough [44], though the relationship between specific maintenance protocols and starter performance cannot be determined from this descriptive data alone.

**Figure 1:**
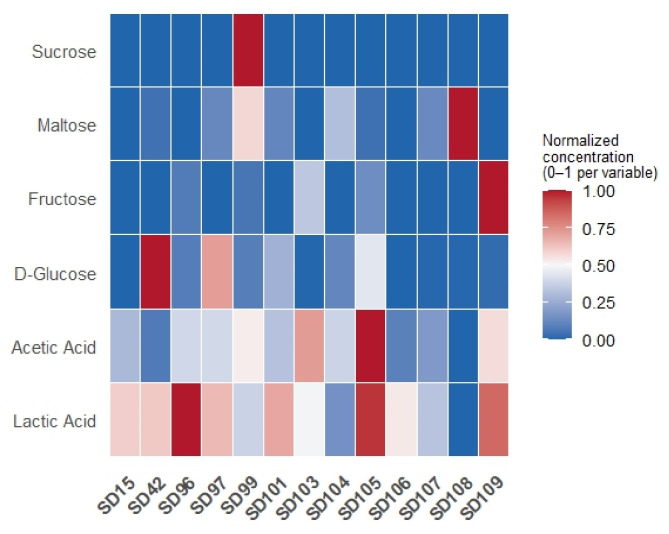
Heatmap of sugar and organic acid concentrations in sourdough samples. Colour scale represents min-max normalized concentrations calculated per variable (0 = minimum, 1 = maximum).

The thirteen sourdoughs analysed showed marked variability in metabolic profiles, with significant differences in sugar consumption and organic acids production, which may have important implications for bread quality (Figure 1, Table S9). The incomplete fermentation of sucrose in SD99 (0.076 ± 0.01 mM), maltose in SD99, SD104 and SD108 (36.94 ± 0.02, 20.33 ± 0.01 and 64.04 ± 0.01 mM, respectively), and fructose in SD103 and SD109 (0.94 ± 0.01 and 2.72 ± 0.01 mM, respectively), likely reflects differences in the microbial composition, metabolic activity and carbon source preferences across sourdoughs [45,46]. Maltose accumulation was the most indicative anomaly: SD108 exhibited the highest residual concentration (64.04 ± 0.01 mM), suggesting reduced maltose catabolism. The mechanistic basis of this failure, whether it is enzymatic, regulatory or community-level, remains to be determined. Meanwhile, SD99 (36.93 ± 0.02 mM) and SD104 (20.33 ± 0.01 mM) showed intermediate levels, which are consistent with incomplete utilisation. Concurrent D-glucose accumulation in SD42 (30.53 ± 0.15 mM) and SD97 (22.26 ± 0.03 mM), further suggests that fermentative limitation in these samples. The depletion of fructose, sucrose and maltose across almost all samples was consistent with the expected metabolic behaviour of the active sourdough consortia and confirms a competitive environment in which both yeasts and lactic acid bacteria quickly utilize simpler sugars before switching to more complex carbon sources. Since maltose, generated by amylolytic hydrolysis of starch, is the primary fermentable substrate in sourdough, its persistence directly affects acidification kinetics and overall fermentative output [47].

Lactic acid concentrations ranged from 32.0 ± 0.13 mM (SD108) to 103.0 ± 0.31 mM (SD96), while acetic acid concentrations ranged from 10.9 ± 0.10 mM (SD108) to 33.5 ± 0.25 mM (SD105), with statistically significant differences between samples for both analytes (*p* < 0.05). The most metabolically anomalous sample, SD108, displayed the lowest concentrations of both lactic acid and acetic acid alongside the highest residual maltose, suggesting a severely compromised fermentative consortium with reduced capacity across all major metabolic axes. Conversely, SD105 exhibited the highest concentrations of both organic acids (lactic acid: 100 ± 0.97 mM; acetic acid: 33.5 ± 0.25 mM) alongside moderate residual glucose (14.15 ± 0.03 mM), suggesting high overall metabolic activity. Despite showing one of the highest lactic acid concentrations (103 ± 0.31 mM), SD96 displayed only moderate acetic acid production (19.86 ± 0.21 mM), consistent with a predominantly homofermentative phenotype. This metabolic diversity was further highlighted by the fermentation quotient (FQ), which ranged from 2.21 (SD104) to 5.93 (SD42). Samples with FQ > 5.0 (namely, SD42, SD106, and SD96) are indicative of predominantly homolactic fermentation [48], characterized by rapid dough acidification and enhanced gluten structuring. Samples with FQ < 3.0 (namely SD104, SD103, SD99, and SD108) indicated a greater heterofermentative component, in which lactic acid, acetic acid, and CO_2_ are produced as parallel metabolic outputs [49]. The co-occurrence of low FQ and high residual maltose in SD104 and SD99 is noteworthy.

This convergent evidence, high substrate residue and low acid output, represents a clear phenotype of fermentative insufficiency. Nevertheless, investigating these outliers at the strain level is crucial to identify the metabolic bottlenecks or the lack of specific enzymatic pathways that prevent efficient sourdough fermentation.

### 3.2. Integrating NGS Sequencing and Culture-Dependent Isolation for a Comprehensive Microbial Analysis

**Figure 2:**
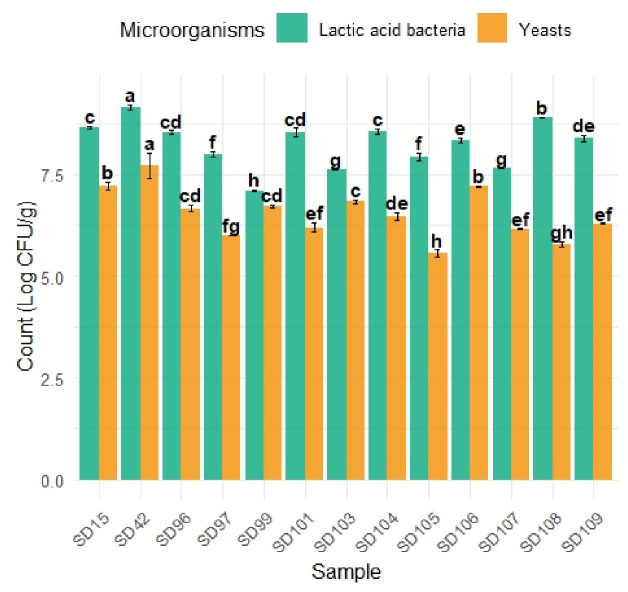
Microbial count on sourdough samples. Different letters indicate statistically significant differences among samples (*p* < 0.05).

Microbial counts revealed that bacterial populations exceeded yeast populations (8.26 ± 0.57 vs. 6.52 ± 0.62 log CFU/g) (Figure 2, Table S10). The LAB-to-yeast ratio varied substantially across samples, ranging from 2:1 (SD99) to 241:1 (SD105). These values fall within the wide range (10:1 to 10,000:1) reported in the literature for traditional sourdoughs [50] and are consistent with 100:1 ratio typically associated with type II sourdough [51,52]. This variability is attributable to differences in microbial populations derived from the source ingredients [6] and is reflected in the fermentative profiles.

Particularly, samples with LAB dominance (SD105, SD101) corresponded to the highest organic acid outputs, while SD99, (LAB-to-yeast ratio:2:1), displayed intermediate acid concentrations and a low fermentation quotient.

**Figure 3:**
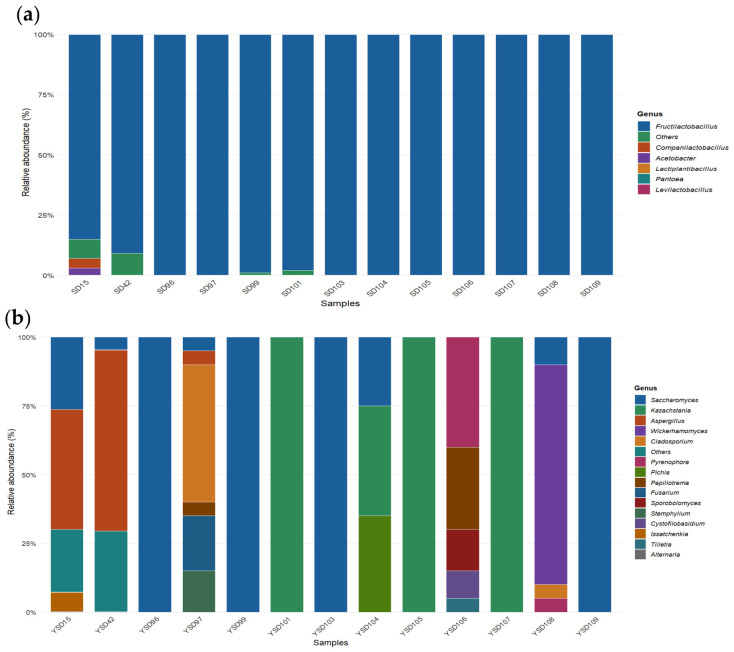
(**a**) Bacterial community composition at genus level in sourdough samples based on 16S rRNA gene sequencing (V3-V4 regions); (**b**) Stacked bar chart showing the relative abundance (%) of fungal genera detected in each sample.

The metabarcoding analysis based on NGS sequencing of the 16S rRNA gene reveals that the bacterial communities are strongly dominated by lactic acid bacteria (Figure 3a). The genus *Fructilactobacillus* was markedly prevalent across all samples, with relative abundances ranging approximately from 93% to 100%. Other genera typical of sourdough ecosystems were detected in lower percentages, including *Lactiplantibacillus* (0.04%), *Levilactobacillus* (0.01%), *Companilactobacillus* (0.27%) and *Acetobacter* (0.21%). Analysis of the fungal composition of sourdough (Figure 3b) reveals marked heterogeneity among the samples, reflecting the different management and maturation conditions of the starter culture. Most samples (SD96, SD99, SD101, SD103, SD105, SD107, SD109) show an exclusive dominance of fermentative yeasts with *Saccharomyces* or *Kazachstania* representing 100% of the assigned fungal community. Sample SD104 exhibited a more diverse fermentative yeast community, with co-occurrence of *Kazachstania* (40%), *Pichia* (35%), and *Saccharomyces* (25%), suggesting an immature or transitional microbiota in which fermentative competence is distributed across multiple yeast genera rather than consolidated under a single dominant taxon. Sample SD106, by contrast, was characterised by a profile dominated by non-fermentative and environmentally associated fungal taxa, including *Pyrenophora* (40%), *Papiliotrema* (30%), *Cystofilobasidium* (10%), and *Tilletia* (5%), with *Saccharomyces* representing only 15% of the ITS reads [53]. Sample SD108 is dominated by *Wickerhamomyces* (80%). Other samples show problematic contamination by filamentous fungi: SD42 is dominated by Aspergillus (65.9%), while SD15 showed a mixed profile mainly composed of Aspergillus (43.68%) and *Saccharomyces* (26.24%). SD97 presents *Cladosporium* (50%) and *Fusarium* (20%).

The richer genus-level diversity identified through metabarcoding-based profiling of the mycobiota has highlighted the ecological complexity of the fungal community, warranting a more thorough culture-dependent characterization. To this end, a total of 130 yeast isolates were obtained from 13 sourdoughs and identified by D1/D2 sequencing and MALDI-TOF mass spectrometry. Among them 58.46% (76 isolates) were identified as *S. cerevisiae*, while the remaining 41.54% belonged to non-*Saccharomyces* species, including *Torulaspora delbrueckii* with 20 isolates (15.38%), *Pichia fermentans* with 12 isolates (9.23%), *Wickerhamomyces anomalus* with 10 isolates (7.69%), *Maudiozyma humilis* with 9 isolates (6.92%), and *Monosporozyma unispora* with 3 isolates (2.31%). These proportions reflect the recoverable, morphologically distinct fraction of the yeast community under the isolation conditions applied and should not be interpreted as quantitative estimates of actual species abundance in the sourdoughs. The identified strains have been deposited in the MBDS-UNISSCC microbial collection of University of Sassari, and the relevant information has been made available via registration in the database (https://www.mbds.it/en/mbds-search/, accessed on 1 December 2025).

A direct comparison of culture-based identification and ITS metabarcoding across all 13 samples revealed substantial and pervasive method-dependent discordance (Table S10). Complete concordance, defined as the dominant genus recovered by culture corresponding to the most abundant genus in the ITS profile, was observed in only 2 out of 13 samples (15.4%): SD99 and SD103, both yielding exclusively *Saccharomyces* by both methods. In the remaining 11 samples (84.6%), at least one form of discordance was recorded. In 9 of these (SD15, SD42, SD96, SD97, SD101, SD104, SD105, SD106, SD108), the dominant genus identified by culture did not correspond to the dominant genus in the ITS profile. The most striking cases include SD96, where 10/10 isolates were identified as *Wickerhamomyces anomalus* by culture yet ITS detected exclusively *Saccharomyces* (100%); SD97, where 10/10 culture isolates were *Torulaspora delbrueckii* whereas ITS showed *Cladosporium* (50%) and *Fusarium* (20%) with no *Torulaspora* signal; and SD42, where culture isolated *S. cerevisiae* exclusively (10/10) while ITS was dominated by *Aspergillus* (65.9%).

These discordances represent a systematic disagreement between the two methodological frameworks. Culture-based isolation selectively recovers viable, metabolically active taxa, whereas culture-independent NGS captures the totality of environmental DNA irrespective of cellular viability, a distinction with far-reaching interpretive consequences. This divergence is particularly consequential in complex fermented ecosystems where *Candida*, *Kazachstania*, and *Wickerhamomyces* spp. frequently co-occur, and where species-level resolution achieved through MALDI-TOF MS does not necessarily correspond to genus-level assignments derived from high-throughput amplicon sequencing [54]. This outcome is consistent with well-documented limitations of amplicon-based sequencing in complex fermented matrices, where taxon-specific biases or the dilution of low-abundance taxa below reliable detection thresholds may prevent the recovery of otherwise viable species [55]. Differential DNA extraction efficiency across yeast taxa constitutes an additional source of bias, as interspecific variation in cell wall architecture directly modulates DNA yield independently of actual cellular abundance [55]. Critically, neither method should be interpreted as validating or correcting the other, as they capture fundamentally different and only partially overlapping windows of mycobiota composition: culture recovers viable, metabolically active cells cultivable under selective conditions, whereas ITS metabarcoding captures total environmental fungal DNA irrespective of viability, cellular integrity, or cultivability. Recent comparative investigations converge on the interpretation that culture-dependent and culture-independent frameworks are best regarded as complementary: their integration affords simultaneous insight into the functionally active microbial fraction and the overall diversity architecture of the fermented ecosystem [56]. Their integrated use is therefore necessary precisely because of their divergent outputs, which together provide a more complete picture of the sourdough fungal community.

The frequent recovery of *S. cerevisiae* by culture-dependent isolation is consistent with it well-known role as the primary leavening agent in sourdough system [57] and with its robust cultivability on WL agar under the conditions applied; whereas non-*Saccharomyces* species may contribute to the development of aroma and flavor through their diverse metabolic activity, as also reported by other authors [58]. Although *Pichia* is less fermentatively efficient than *Saccharomyces*, its presence (35% in SD104) contributes to the development of complex aromatic profiles (De Vuyst et al., 2016) [45]. The detection of *Maudiozyma humilis* (6.92%), an obligately maltose-negative yeast that relies on glucose released by heterofermentative LAB as its primary carbon source [59], can explain the maltose accumulation observed in certain samples. Where this species contributes significantly to fungal biomass, maltose may remain underutilised by the yeast fraction; however, the relative contribution of community composition versus strain-level regulatory mechanisms such as glucose catabolite repression cannot be resolved from the current data alone. *Torulaspora delbrueckii* (15.38%) and *Wickerhamomyces anomalus* (7.69%) are expected to contribute to aromatic complexity and microbiological stability respectively [60,61], while *Pichia fermentans* (9.23%) likely enriches the volatile fraction via the Ehrlich pathway. Overall, the observed variability in fermentative performance across samples reflects the integrated metabolic activity of a compositionally diverse consortium, consistent with evidence that sourdough functional properties cannot be predicted from the physiology of a single species alone [62].

The detection of *Aspergillus* (65.9% in SD42) and *Fusarium* (20% in SD97) in ITS metabarcoding profiles warrants consideration from a food safety perspective, as these genera include mycotoxin-producing species in cereal-based systems [63]. The occurrence of filamentous fungal DNA in sourdough ITS profiles has been reported previously, including the detection of *Fusarium culmorum*, *Fusarium petersiae*, *Alternaria tenuissima*, and *Microdochium seminicola* across spontaneous sourdough propagation cycles [64]. However, amplicon-based detection of these taxa does not constitute direct evidence of mycotoxin contamination, as non-viable or environmental fungal DNA may contribute to metabarcoding signals independently of active growth. Furthermore, mycotoxin levels in sourdough are dependent on toxin type, LAB strain, and process conditions, with LAB-mediated reduction reported for deoxynivalenol [65] but not for ochratoxin A [66]. These findings therefore identify targeted mycotoxin analysis and flour quality assessment as priorities for future work.

Although MALDI-TOF MS has demonstrated high identification success rates, making it a reliable and advantageous tool for yeast identification, its accuracy depends heavily on the comprehensiveness of the reference database. In the present study, MALDI-TOF MS and D1/D2 sequencing yielded concordant species-level identifications for 125 out of 130 isolates (96.2%). The five discordant cases (3.8%) were resolved by ITS sequencing as a third arbitrating method: two isolates showed D1/D2 misidentification attributable to colony contamination during subculturing (final identification confirmed by both MALDI-TOF MS and ITS); one isolate exhibited an inter-method conflict resolved by ITS; and two isolates produced ambiguous MALDI-TOF spectral profiles that could not distinguish between two database entries (DBAL-13.0), with ITS confirming the D1/D2 result in both cases (see Supplementary Table S11). Several factors can influence spectral quality and reproducibility, including culture conditions, sample preparation protocols, matrix selection and strain biological variability [67,68]. Furthermore, the score thresholds that have been established for bacterial identification may not be optimal for yeast identification. Some studies have suggested that a cut-off value of 1.70, rather than the manufacturer-recommended value of 2.00, is more effective for correctly identifying yeast species [69,70]. The concordance rate obtained here (96.2%) with a threshold of ≥2.00 is consistent with these findings and supports the use of a slightly relaxed cut-off when MALDI-TOF MS is applied in combination with a molecular reference method.

### 3.3. Biotyping of S. cerevisiae Strains Underlines a Phylogenetically Heterogeneous Population

As the dominant species detected in this study, a total of 76 *Saccharomyces cerevisiae* strains, isolated from 10 sourdough samples (SD15, SD42, SD99, SD101, SD103, SD104, SD105, SD107, SD108, SD109), were subjected to biotyping and subsequently compared with the profiles of 43 reference strains of known origin (Table S7).

**Figure 4:**
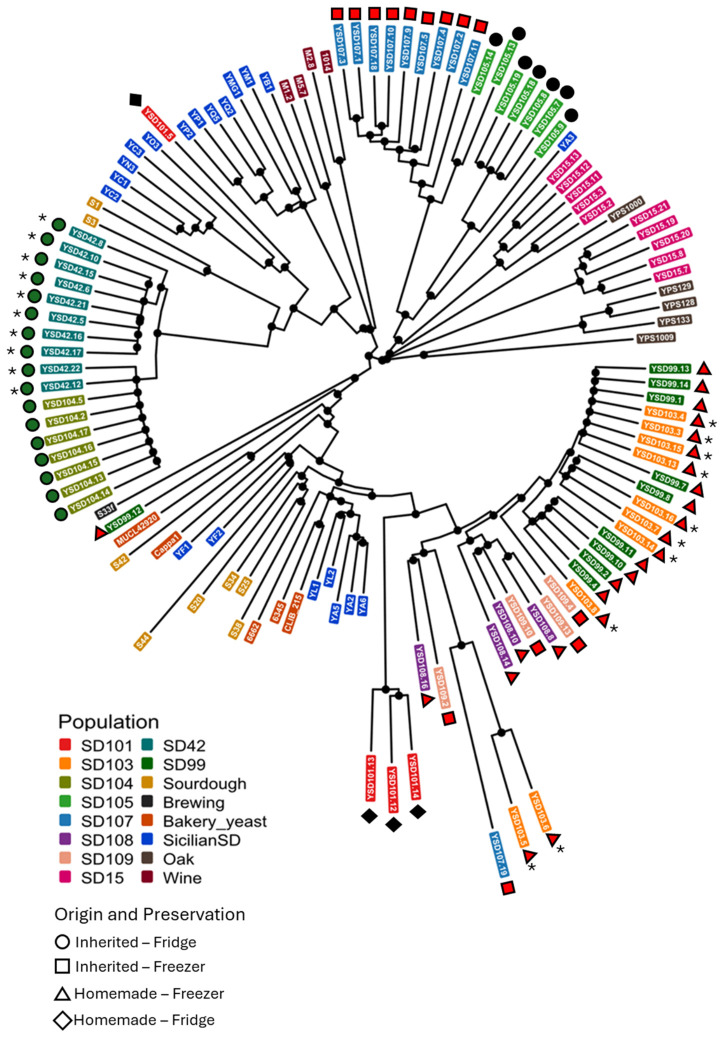
Phylogenetic tree of Saccharomyces cerevisiae strains based on microsatellite data and a genetic distance matrix. Oak-associated strains were used as outgroup to root the tree. Bootstrap values are not reported; robustness of major group differentiation was assessed by iterative removal of single loci, confirming the stability of major genetic group separation across locus subsets. Maintenance metadata are encoded for each isolate through symbol shape and fill colour. Symbol shape indicates starter provenance and storage method: ○—inherited, refrigerator storage; □—inherited, freezer storage; △—homemade, freezer storage; ◇—homemade, refrigerator storage. Symbol fill colour indicates refreshment frequency: black—daily; green—weekly; red—as needed. Asterisks (*) denote starters used to produce traditional bread typologies; their absence indicates exclusive production of common bread.

A microsatellite-based phylogenetic analysis revealed a hierarchically structured genetic diversity among *S. cerevisiae* isolates (Figure 4, the raw data, including the allele table and distance matrix, are available in the Supplementary Materials Tables S12 and S13). This reflects multiple, independent domestication trajectories, as visualized in the consensus neighbour-joining tree rooted in oak-associated strains, which were selected as an outgroup due to their genetic distance from anthropized fermentative populations. Within the internally branched portion of the tree, several independent SD lineages were resolved. A major, well-supported cluster grouped the isolates from SD99, SD101, SD103, SD108, and SD109 sourdough samples with the industrial reference strains (CLIB 215, MUCL 42920), commercial baking strains (Lesaffre Cappa1, Lallemand 6662 and 6345), and Sicilian sourdough isolates (YA5, YL1, YA2, YA3), characterized by minimal microsatellite divergence and tri- to tetraploid genotypes.

This suggests either shared ancestry or strong convergent selection in human-associated fermentative environments. This finding extends the results obtained by Marongiu et al. [71] for Sardinian bakery’s yeasts, who identified a major cluster of tetraploid bread strains grouping isolates from France, Japan, Sicily, and Spain [39,72]. It is also consistent with the broader pattern of technology-driven *S. cerevisiae* diversification documented on a global scale [57]. A neighbouring but clearly distinct cluster comprised SD42 and SD104 isolates, grouping closely with Sicilian sourdough strains (YC1, YC2) while remaining genetically dissimilar. This indicates a shared genetic background, followed by local diversification. Notably, SD42 and SD104 strains displayed similarly low intra-cluster diversity, a pattern also observed among SD99 strains. SD99 and SD103 strains co-clustered despite originating from independent households, while SD103 isolates displayed increased internal genetic heterogeneity. The isolates from sourdough SD107 formed one of the most compact and genetically stable clusters in the dendrogram, showing very limited intra-cluster variability. In contrast, the closely adjacent but clearly separated SD105 branch supports the existence of related yet independent evolutionary trajectories, distinct from both the industrial-proximal and the ancestral clades. Most notably, the SD15 isolates formed two well-defined, isolated clusters, distant from both the industrial-related and sourdough-associated groups. These two clusters were connected closer to the ancestral root, represented here by the wild oak-associated reference strains (YPS128, YPS129, YPS133, YPS1009), suggesting the presence of two other domesticated lineage that should be verified using genomic data. SD99, SD103, SD108, and SD109 isolates occupied positions within the industrial-proximal cluster, and these strains contained three or four alleles at several loci, in a similar manner to the industrial bread starters of this cluster. The co-occurrence of brewing and bakery reference strains within this group is consistent with findings of Marongiu et al. [71], who observed that the commercial brewing strain Safbrew-S33 clusters within the bread strain group, reflecting a partially shared domestication trajectory between baker’s and brewer’s lineages.

To explore whether, in a descriptive framework, maintenance practices show any apparent association with genetic proximity among sourdough-associated *S. cerevisiae* strains, the metadata collected for each starter (storage conditions, refreshment frequency, and bread typology) overlaid onto the microsatellite-based phylogenetic tree.

No consistent association was observed between maintenance practices and phylogenetic clustering. Within the large industrial-proximal cluster, grouping isolates from SD99, SD101, SD103, SD108, and SD109, starters maintained under heterogeneous conditions coexist: SD99, SD103, SD108 and SD109 are stored in the freezer and refreshed as needed, while SD101 is stored in the fridge and refreshed daily. Despite these differences, all occupy equivalent phylogenetic positions within the cluster. Notably, SD103 is the only starter within this cluster used to produce traditional bread types alongside common bread, yet this distinctive use is not reflected in any phylogenetic separation from the remaining industrial-proximal isolates. SD42 and SD104, which co-cluster with low intra-population variability, share inherited origin, refrigerator storage, and weekly refreshment. SD42 is also notably used to produce traditional bread together with SD103. Yet despite this shared cultural role, SD42 and SD103 occupy distinct phylogenetic positions, further illustrating the absence of a detectable association between bread typology and genetic clustering. SD107 and SD105, which form sister taxa, present contrasting profiles: SD107 is inherited, stored in the freezer and refreshed as needed, while SD105 is inherited, stored in the refrigerator and refreshed daily. For SD15, no maintenance metadata are available, precluding any comparison. Overall, the distribution of maintenance practices and bread typologies across the tree does not follow phylogenetic structure.

The inter-delta fingerprinting analysis (Figure S1) was broadly congruent with the microsatellite-based phylogeny for most of the groups examined. High concordance was observed for SD107, SD105, SD42, and SD99, which formed cohesive, well-supported clusters in both analyses, confirming the robustness of these lineages across independent typing methods. Notably, SD15 displayed consistent internal bipartition in both approaches, with isolates YSD15.19 and 21 forming a distinct subcluster separate from the remaining SD15 strains, a subdivision independently captured by both retrotransposon fingerprinting and microsatellite-based phylogenetic reconstruction. Partial concordance was observed for SD104, SD103, SD108, SD109, and SD101, which shared the same broad phylogenetic neighbourhood in the Neighbor-Joining (NJ) tree but showed variable internal resolution between methods. Specifically, SD103 appeared as a compact cluster in the inter-delta dendrogram yet was paraphyletic in the microsatellite-based tree, suggesting the presence of internal genetic heterogeneity not fully resolved by retrotransposon fingerprinting. Similarly, SD108 and SD109 were indistinguishable by inter-delta analysis but co-localised with SD101, SD103, and SD99 in the NJ tree without forming a discrete clade, while SD101 was erroneously placed near SD105 by inter-delta analysis despite occupying a distinct phylogenetic position adjacent to SD108 and SD109 in the microsatellite tree.

These discordances are consistent with the lower discriminatory power of retrotransposon-based fingerprinting relative to microsatellite typing, which remains the higher-resolution reference for intraspecific strain discrimination in *S. cerevisiae*.

### 3.4. Biotyped T. delbrueckii Strains Set Represent an Independent Lineage?

As the second most frequently identified species in this study, with 20 isolates identified, we sought to investigate this further by biotyping the *T. delbrueckii* isolates, as previously done for *S. cerevisiae*.

**Figure 5:**
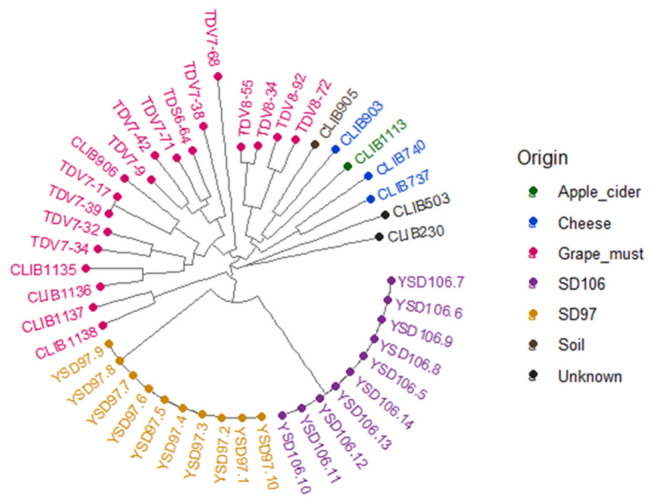
Dendrogram of *Torulaspora delbrueckii* strains from Sardinian sourdoughs (YSD106, YSD97) and CIRM-Levures references, clustered by microsatellite analysis using Dc Chord distance. Bootstrap values are not reported, as multiple strains share identical multilocus genotypes whose grouping reflects arithmetic identity rather than phylogenetic inference.

The microsatellite-based phylogenetic tree (Figure 5, raw data are available in the Supplementary Materials Tables S14 and S15) revealed a clear genetic structuring among *T. delbrueckii* isolates. Notably, the SD97 and SD106 isolates were completely separated from all available reference strains, including those from grapes, cheese, soil, and cider for a total of 26 reference strains (Table S8).

Strains from SD97 and SD106 sourdoughs formed two distinct clusters of the tree, with no close phylogenetic counterparts in existing collections. This may suggest that these isolates represent previously uncharacterized lineages endemic to the local sourdough tradition. While the two clusters share a common basal node, they remain clearly distinct from one another, indicating a shared ancestral origin followed by independent divergence, consistent with prolonged maintenance through backslopping in separate households.

This phylogenetic positioning acquires additional interpretive depth considering the pangenomic framework recently established for *T. delbrueckii*, which identifies five major clades within the species, three of European origin [73]. Among these, a wild arboreal lineage is a sister lineage to two anthropic clades: one associated with wine fermentation and one, designated Mixed-Anthropic, linked to diverse human-associated environments including dairy and bread dough. The separation of SD97 and SD106 from all documented reference strains, including Mixed-Anthropic isolates, raises the question of their precise placement within the evolutionary framework proposed by Silva et al. [73]. A comparative analysis including *T. delbrueckii* strains specifically isolated from sourdough would be necessary to determine whether the Sardinian isolates described here represent a distinct branch within the Mixed-Anthropic clade or an independent lineage, and to clarify their genomic adaptations to the bread fermentation niche.

### 3.5. Harnessing Citizen Data for Sourdough Microbial Resource Innovation

The quality of the biological material collected within the TRIGU framework was supported by a structured sampling protocol communicated to participants via a dedicated vademecum prepared by the research team and distributed through the Municipality of Villaurbana. Participants were instructed to collect sourdough samples in sterile containers and to include a portion of the flour used for the most recent refreshment cycle; samples were transported under refrigerated conditions on the day of collection to minimize alterations to the native microbial composition prior to laboratory processing. Despite this framework, metadata remained incomplete for SD15, reflecting the inherent variability in participant engagement that characterizes citizen science initiatives and constituting a recognized limitation of the present dataset. Community engagement extended beyond sample collection: two public dissemination events were organized in which the research team presented preliminary and final results directly to sourdough owners, establishing a bidirectional exchange between scientific and community knowledge consistent with best practices in participatory research [16,18].

The integrated approach utilized for the characterization of the studied sourdoughs provides the basis for the selection of candidate strains to be evaluated in future experimental works as part of the Municipality of Villaurbana outcomes of TRIGU project. The *S. cerevisiae* component of the proposed candidate pool can be drawn from four phylogenetically distinct populations (SD107, SD15, SD42, and SD15) selected to represent the breadth of the genetic diversity of the sourdoughs collected in Villaurbana and excluding strains phylogenetically related to commercial baker’s yeast. Given the absence of associations between maintenance practices and phylogenetic clustering, strain selection was based on phylogenetic coherence and NGS community profiles. SD107 (recommended strains: YSD107.10, YSD107.5, YSD107.11, YSD107.2, YSD107.1) showed the most internal compact cluster and near-exclusive *Saccharomyces* dominance in NGS profiling. SD105 (recommended strains: YSD105.7, YSD105.9, YSD105.14) occupies a phylogenetically independent position with comparable yeast dominance. *S. cerevisiae* strains recovered from the SD42 sourdough (recommended strains: YSD42.6, YSD42.8, YSD42.22), despite *Issatchenkia* dominance in NGS profiling, showed internal genetic coherence; their potential inclusion in a future consortium design may confer resilience traits of relevance under discontinuous maintenance regimes, a hypothesis to be assessed through co-culture experiments and backslopping cycles. SD15 (candidate strains: YSD15.7, YSD15.11, YSD15.13, YSD15.19, YSD15.20, YSD15.21) exhibits the greatest phylogenetic divergence within the dataset, with extended branch lengths reflecting an independent evolutionary trajectory. The mixed NGS community profile of SD15 and the absence of co-culture data require experimental verification of competitive behaviour prior to any inclusion in a consortium design.

The non-*Saccharomyces* component of the candidate pool is represented by *Torulaspora delbrueckii*, which accounted for 15.38% of total isolates and constitutes the second most frequent species identified. Isolates YSD97 and YSD106 define two clonal lineages with no phylogenetic overlap with globally characterized reference strains from grape, cheese, soil, or cider substrates, representing previously undescribed endemic Sardinian diversity. These isolates are proposed as candidates for experimental validation; if confirmed in co-culture and model fermentation assays, their inclusion would introduce ester and higher alcohol profiles complementary to the leavening activity of *S. cerevisiae*, while their biogeographic specificity would anchor the consortium to the fermentation heritage of Villaurbana. The bacterial component remains to be fully characterized. However, NGS profiling consistently assigns the dominant lactic acid bacteria to the genus *Fructilactobacillus*, which will be the subject of dedicated isolation and characterization in subsequent works.

Experimental validation of the candidate consortium will require co-culture assays to assess both synergistic and antagonistic interactions among all constituent strains and competitive dynamics, simulated backslopping cycles to evaluate stability across successive fermentation rounds, model bread fermentations to assess technological performance, and calibration of fermentation parameters under Type IV protocols. This validation framework applies to the entire candidate pool, as inter-strain interactions cannot be predicted from individual strain characterization alone regardless of phylogenetic origin. Based on the TRIGU metadata and established parameters for Type IV sourdoughs [74], fermentation temperatures between 20 and 30 °C are recommended to support the metabolic stability of *Fructilactobacillus* spp. and the competitive viability of both *S. cerevisiae* and *T. delbrueckii*. Particularly, temperatures exceeding 30 °C suppress yeast activity in favor of rapid bacterial acidification and should be avoided.

## 4. Conclusions and Perspectives

The TRIGU citizen science project demonstrates that the active involvement of a rural community in the documentation and biological preservation of traditional sourdough starters can generate microbiological data of scientific and biotechnological value. Despite originating from a single municipality, the thirteen sourdoughs exhibited remarkable fermentative and genetic diversity. This reflects the coexistence of both homofermentative and heterofermentative metabolic strategies driven by differences in management practices and backslopping protocols.

The characterisation of the yeast community revealed significant *S. cerevisiae* intraspecific diversity and the segregation of *T. delbrueckii* strains into two clonal groups substantially divergent from reference strains, documenting a possible endemic Sardinian genetic diversity. Taken together, these data confirm that yeast genetic diversity operates at multiple levels, from species composition to intraspecific strain structure, encompassing ancestral, endemic, and industrially influenced lineages, and underscoring the value of traditional sourdough collections as repositories of biotechnological diversity. The long-term valorization and protection of these microbial resources is ensured by their deposition in certified biological collections such as MBDS-UNISSCC, in accordance with the FAIR principles. The characterisation data presented here forms the foundation for the design of a representative sourdough microbial consortium to be proposed to the Municipality of Villaurbana, with candidate strains from SD107, SD105, SD42, and SD15 for the *S. cerevisiae* component and from YSD97 and YSD106 for *T. delbrueckii*, as the basis for a locally anchored, reproducible and customised sourdough pending experimental validation of their co-culture compatibility and technological performance.

Future research priorities should include the full characterization of the bacterial component, currently assigned by NGS profiling to the genus *Fructilactobacillus*, through dedicated culture-dependent isolation and subsequent genetic and phenotypic analyses. Experimental validation of the proposed consortium under Type IV fermentation protocols will be required to assess competitive dynamics among *S. cerevisiae*, *T. delbrueckii*, and *Fructilactobacillus* spp. and to calibrate maintenance parameters for artisanal production. Expansion of the citizen science approach to other Sardinian municipalities, combined with the extension of MALDI-TOF MS reference databases to food-relevant species of bacteria and yeasts, will broaden the geographic and taxonomic resolution of traditional sourdough biodiversity.

## Figures and Tables

**Table 1:** Survey data on sourdoughs maintenance protocols from Villaurbana households (n = 13), showing variation in origin, first fermentation conditions, backslopping frequency, storage methods and bread typologies.

Number Catalogue SD	Origin	Flour Used for Dough Preparation	Temperature and Time of Fermentation	Frequency of Backslopping	Storage	Bread Typology
SD15	ND	ND	ND	ND	ND	ND
SD42	inherited	durum wheat semolina	10–12 h Room Temperature	weekly	Fridge	Traditional bread, Bread with gerdas *
SD96	homemade	durum wheat semolina	10 h Room Temperature	as needed	Freezer	Common bread
SD97	inherited	durum wheat semolina	12 h at 18 °C	as needed	Freezer	Common bread, pizza
SD99	homemade	durum wheat semolina	24 h Room Temperature	as needed	Freezer	Common bread
SD101	homemade	durum wheat semolina	3 h Room Temperature	daily	Fridge	Common bread, sweets
SD103	homemade	durum wheat semolina	3 h Room Temperature	as needed	Freezer	Common bread, Traditional bread
SD104	inherited	durum wheat semolina	12 h at 18 °C	weekly	Fridge	Common bread
SD105	inherited	ND	10 h Room Temperature	daily	Fridge	Common bread
SD106	inherited	durum wheat semolina	48 h Room Temperature 8–10 °C	as needed	Freezer	Common bread
SD107	inherited	durum wheat semolina	24 h Room Temperature	as needed	Freezer	Common bread
SD108	homemade	durum wheat semolina	24 h Room Temperature	as needed	Freezer	Common bread
SD109	inherited	durum wheat semolina	24 h Room Temperature	as needed	Freezer	Common bread

* Gerdas = rendered pork fat cracklings incorporated into the bread dough.

**Table 2:** pH, Total Titratable Acidity (TTA) and Dough Yield (DY) of sourdoughs. Values are expressed as mean ± standard deviation (SD).

Sample	pH Value	TTA (mL NaOH 0.1 M)	DY
SD42	4.64 ± 0.01 ^a^	15.0 ± 0.00 ^ab^	150
SD101	4.55 ± 0.02 ^b^	8.7 ± 0.00 ^de^	150
SD99	4.39 ± 0.01 ^c^	9.0 ± 0.70 ^de^	160
SD97	4.37 ± 0.02 ^c^	14.2 ± 0.85 ^abc^	150
SD15	4.36 ± 0.09 ^c^	15.0 ± 1.48 ^ab^	200
SD103	4.29 ± 0.00 ^d^	8.6 ± 1.48 ^e^	150
SD96	4.27 ± 0.03 ^d^	17.1 ± 0.85 ^a^	200
SD109	4.25 ± 0.01 ^d^	11.8 ± 0.49 ^bcde^	200
SD104	4.23 ± 0.00 ^d^	11.2 ± 0.21 ^cde^	135
SD105	4.16 ± 0.03 ^e^	14.7 ± 1.56 ^ab^	200
SD106	4.12 ± 0.02 ^e^	11.0 ± 0.14 ^cde^	150
SD108	4.11 ± 0.01 ^ef^	10.4 ± 0.00 ^de^	200
SD107	4.06 ± 0.01 ^f^	12.0 ± 0.14 ^bcd^	150

Values within the same column followed by different lowercase letters are significantly different (*p* < 0.05).

## Data Availability

Raw 16S rRNA gene and ITS amplicon sequencing reads have been deposited in the European Nucleotide Archive under accession number PRJEB114392. The ASV/OTU tables, per-sample QC summaries, and DADA2 read-tracking tables for both 16S and ITS datasets are provided as Supplementary Materials. The same applies to the allele tables and distance matrices used to construct phylogenetic trees for isolates of *S. cerevisiae* and *T. delbrueckii*.

## Supplementary Materials

The following supporting information can be downloaded at: https://www.mdpi.com/article/10.3390/foods15132307/s1, Table S1. ASV table 16S; Table S2. ASV table ITS; Table S3. DADA2_16S_read_summary; Table S4. DADA2_ITS_read_summary; Table S5. DADA2_read tracking 16S; Table S6. DADA2_read tracking ITS; Table S7. *Saccharomyces cerevisiae* reference strains used for biotyping comparison, geographical origin and isolation source; Table S8. *T. delbrueckii* reference strains used for biotyping comparison, their geographical origin and isolation source; Table S9. Sugar and organic acid composition of 13 sourdough samples (SD); Table S10. Comparison of cultivable yeast isolates and fungal relative abundances determined by ITS metabarcoding in sourdough samples; Table S11. Yeast isolate identification by D1/D2 sequencing, MALDI-TOF MS, and ITS sequencing; Table S12. Allele table *S. cerevisiae*; Table S13. Distance Matrix *S. cerevisiae;* Table S14. Allele table *T. delbrueckii*; Table S15. Distance Matrix *T. delbrueckii*; Figure S1. Dendrogram obtained from inter-delta PCR profiles of *Saccharomyces cerevisiae* isolates, generated using the Dice correlation coefficient with a tolerance of 1.5, showing the genetic relationships among the analysed strains.

## Abbreviations

The following abbreviations are used in this manuscript:

NGS	Next Generation Sequencing
ASV	Amplicon Sequence Variants
MALDI-TOF MS	Matrix-Assisted Laser Desorption/Ionization Time-Of-Flight Mass Spectrometry
PCR	Polymerase Chain Reaction
NJ	Neighbor-Joining
BLAST	Basic Local Alignment Search Tool
LSU	Large Subunit
ITS	Internal Transcribed Spacer
LRT	Long Terminal Repeats
HCCA	α-cyano-4-hydroxycinnamic acid
UPGMA	Unweighted Pair Group Method with Arithmetic Mean
FAIR	Findable, Accessible, Interoperable, Reusable
SOP	Standard Operation Procedures
