# Supplementary material for: Exploring Microbial Diversity and Yeast Typing in Traditional Sourdoughs from Villaurbana (Sardinia, Italy) Using an Integrated Approach
Source: Foods. 2026 Jun 29;15(13):2307. doi: 10.3390/foods15132307 (PMC13361756; doi:10.3390/foods15132307)
Supplement: Supplementary file 1 [file foods-15-02307-s001.zip › Supplementary materials.pdf]

Table S7. *Saccharomyces cerevisiae* reference strains used for biotyping comparison, geographical origin and isolation source.

Strain	Country	Isolation Year	Isolation Source	Collection
1014	Italy – Tuscany	< 2009	wine	Univ. Florence
M1.2	Italy – Tuscany	< 2009	wine	Univ. Florence
M2.8	Italy – Tuscany	< 2009	wine	Univ. Florence
M5.7	Italy – Tuscany	< 2009	wine	Univ. Florence
6345	France	< 2003	bakery yeast	Lallemand
6662	France	< 2003	bakery yeast	Lallemand
Cappa1	France	< 1994	bakery yeast	SPO
CLIB 215	New Zealand	1994	bakery yeast	CIRM
MUCL42920	Morocco	2000	bakery yeast	MUCL
S-33	Italy	—	brewing strain	Laesaffre
S1	Italy – Sardinia	< 2005	sourdough (Marmilla region)	MBDS-UNISSCC
S3	Italy – Sardinia	< 2005	sourdough (Marmilla region)	MBDS-UNISSCC
S20	Italy – Sardinia	< 2005	sourdough (Mejlogu region)	MBDS-UNISSCC
S25	Italy – Sardinia	< 2005	sourdough (Mejlogu region)	MBDS-UNISSCC
S34	Italy – Sardinia	< 2005	sourdough (Mejlogu region)	MBDS-UNISSCC
S38	Italy – Sardinia	< 2005	sourdough (Mejlogu region)	MBDS-UNISSCC
S42	Italy – Sardinia	< 2005	sourdough (Mejlogu region)	MBDS-UNISSCC
S44	Italy – Lombardy	< 2005	sourdough	MBDS-UNISSCC
YA2	Italy – Sicily	< 2004	sourdough	CIRM-Levures
YA3	Italy – Sicily	< 2004	sourdough	CIRM-Levures
YA5	Italy – Sicily	< 2004	sourdough	CIRM-Levures
YA6	Italy – Sicily	< 2004	sourdough	CIRM-Levures
YB1	Italy – Sicily	< 2004	sourdough	CIRM-Levures
YC1	Italy – Sicily	< 2004	sourdough	CIRM-Levures
YC2	Italy – Sicily	< 2004	sourdough	CIRM-Levures
YC3	Italy – Sicily	< 2004	sourdough	CIRM-Levures
YF1	Italy – Sicily	< 2004	sourdough	CIRM-Levures
YF2	Italy – Sicily	< 2004	sourdough	CIRM-Levures
YL1	Italy – Sicily	< 2004	sourdough	CIRM-Levures
YL2	Italy – Sicily	< 2004	sourdough	CIRM-Levures
YM1	Italy – Sicily	< 2004	sourdough	CIRM-Levures

YMG1	Italy – Sicily	< 2004	sourdough	CIRM-Levures
YN3	Italy – Sicily	< 2004	sourdough	CIRM-Levures
YO3	Italy – Sicily	< 2004	sourdough	CIRM-Levures
YP1	Italy – Sicily	< 2004	sourdough	CIRM-Levures
YP2	Italy – Sicily	< 2004	sourdough	CIRM-Levures
YQ2	Italy – Sicily	< 2004	sourdough	CIRM-Levures
YQ5	Italy – Sicily	< 2004	sourdough	CIRM-Levures
YPS1000	USA – New Jersey	2000	oak	SPO
YPS1009	USA – New Jersey	2000	oak	SPO
YPS128	USA – Pennsylvania	1999	oak	SPO
YPS129	USA – Pennsylvania	1999	oak	SPO
YPS133	USA – Pennsylvania	1999	oak	SPO

Table S8. *T.delbrueckii* reference strains used for biotyping comparison, their geographical origin and isolation source.

Strain name	Country	Year	Isolation source	Collection
**CLIB1113**	France - Cote d'Armor	1973	apple cider	CIRM-Levures
**CLIB230**	unknown	unknown	unknown	CIRM-Levures
**CLIB503**	unknown	unknown	gum in sugar refinery	CIRM-Levures
**CLIB737**	France - Aquitaine	2001	cheese	CIRM-Levures
**CLIB740**	France - Haute Savoie	2001	cheese	CIRM-Levures
**CLIB903**	Italy -Sardinia	1964	cheese	CIRM-Levures
**CLIB905**	Sweden	1958	soil	CIRM-Levures
**CLIB1135**	France -Cote d'Or	2007	grape must	CIRM-Levures
**CLIB1136**	France -Cote d'Or	2007	grape must	CIRM-Levures
**CLIB1137**	France -Cote d'Or	2007	grape must	CIRM-Levures
**CLIB1138**	France -Cote d'Or	2007	grape must	CIRM-Levures
**CLIB906**	Switzerland - Valais	1924	grape must	CIRM-Levures
**TDS6-64**	France - Aude	2016	grape must	UMR SPO
**TDV7-17**	France - Aude	2016	grape must	UMR SPO
**TDV7-32**	France - Aude	2016	grape must	UMR SPO

**TDV7-34**	France - Aude	2016	grape must	UMR SPO
**TDV7-38**	France - Aude	2016	grape must	UMR SPO
**TDV7-39**	France - Aude	2016	grape must	UMR SPO
**TDV7-42**	France - Aude	2016	grape must	UMR SPO
**TDV7-68**	France - Aude	2016	grape must	UMR SPO
**TDV7-71**	France - Aude	2016	grape must	UMR SPO
**TDV7-9**	France - Aude	2016	grape must	UMR SPO
**TDV8-34**	France - Gard	2016	grape must	UMR SPO
**TDV8-55**	France - Gard	2016	grape must	UMR SPO
**TDV8-72**	France - Gard	2016	grape must	UMR SPO
**TDV8-92**	France - Gard	2016	grape must	UMR SPO

Table S9. Sugar and organic acid composition of 13 sourdough samples (SD).

Sample	D-Glucose (mM)	Fructose (mM)	Maltose (mM)	Sucrose (mM)	D+L Lactic Acid (mM)	Acetic Acid (mM)
**SD15**	1.05 ± 0 ^{i}	0.00 ^{f}	0.00 ^{i}	0.00 ^{b}	74.50 ± 1.74 ^{e}	17.70 ± 1.50 ^{ef}
**SD42**	30.53 ± 0.15 ^{a}	0.00 ^{f}	2.95 ± 0.02 ^{g}	0.00 ^{b}	75.40 ± 0.19 ^{de}	12.70 ± 1.80 ^{gh}
**SD96**	3.55 ± 0.01 ^{f}	0.22 ± 0.01 ^{d}	0.00 ^{i}	0.00 ^{b}	103.00 ± 0.30 ^{a}	19.90 ± 0.20 ^{de}
**SD97**	22.26 ± 0.03 ^{b}	0.00 ^{f}	8.15 ± 0.06 ^{e}	0.00 ^{b}	78.20 ± 0.47 ^{cd}	19.90 ± 0.04 ^{de}
**SD99**	3.66 ± 0 ^{f}	0.17 ± 0 ^{e}	36.93 ± 0.02 ^{b}	0.76 ± 0.01 ^{a}	59.0 ± 0.27 ^{g}	22.80 ± 0.17 ^{cd}
**SD101**	9.05 ± 0.02 ^{d}	0.00 ^{f}	7.57 ± 0.01 ^{f}	0.00 ^{b}	81.40 ± 0.06 ^{c}	18.20 ± 0.08 ^{ef}
**SD103**	1.17 ± 0.01 ^{hi}	0.94 ± 0.01 ^{b}	0.00 ^{i}	0.00 ^{b}	66.80 ± 1.28 ^{f}	27.30 ± 3.71 ^{b}
**SD104**	4.55 ± 0.01 ^{e}	0.00 ^{f}	20.33 ± 0.01 ^{c}	0.00 ^{b}	43.20 ± 0.33 ^{i}	19.60 ± 0.45 ^{de}
**SD105**	14.15 ± 0.03 ^{c}	0.39 ± 0.01 ^{c}	2.57 ± 0.01 ^{h}	0.00 ^{b}	100.00 ± 0.97 ^{a}	33.50 ± 0.25 ^{a}
**SD106**	1.05 ± 0.01 ^{i}	0.00 ^{f}	0.00 ^{i}	0.00 ^{b}	69.90 ± 0.73 ^{f}	13.20 ± 0.35 ^{gh}
**SD107**	1.22 ± 0 ^{h}	0.00 ^{f}	8.38 ± 0.01 ^{d}	0.00 ^{b}	55.60 ± 0.33 ^{h}	15.20 ± 0.17 ^{fg}
**SD108**	1.22 ± 0 ^{h}	0.00 ^{f}	64.04 ± 0.01 ^{a}	0.00 ^{b}	32.00 ± 0.12 ^{j}	10.90 ± 0.09 ^{h}
**SD109**	1.72 ± 0 ^{g}	2.72 ± 0.01 ^{a}	0.00 ^{i}	0.00 ^{b}	92.2 ± 2.94 ^{b}	23.60 ± 0.33 ^{bc}

Values are expressed as mean ± standard deviation (mM). Where no standard deviation is reported, the value did not vary across replicates. [b] Different lowercase superscript letters within each column indicate statistically significant differences among samples ($p < 0.05$) as determined by multiple comparison testing.

Table S10. Comparison of cultivable yeast isolates and fungal relative abundances determined by ITS metabarcoding in sourdough samples.

Sample	LAB (Log CFU/g)	Yeast (Log CFU/g)	Identified Isolates (culture-method)	Relative Aboundance ITS (%)
**SD15**	8.65 ± 0.04 [c]	7.22 ± 0.09 [b]	10/10 *S. cerevisiae*	*Saccharomyces 26.24*<br>*Aspergillus 43.68*<br>*Issatchenkia 6.99*<br>*Others 22.72*
**SD42**	9.15 ± 0.07 [a]	7.72 ± 0.31 [a]	10/10 *S. cerevisiae*	*Saccharomyces 4.54; Aspergillus 65.9; Others 29.18*
**SD96**	8.53 ± 0.04 [cd]	6.67 ± 0.07 [cd]	*10/10 W. anomalus*	*Saccharomyces 100*
**SD97**	8.01 ± 0.07 [f]	6.00 ± 0.06 [fg]	*10/10 T.delbrueckii*	*Cladosporium 50; Fusarium 20; Stemphylium 15; Papiliotrema 5; Saccharomyces 5; Aspergillus 5*
**SD99**	7.11 ± 0.02 [h]	6.71 ± 0.03 [cd]	*10/10 S. cerevisiae*	*Saccharomyces 100*
**SD101**	8.53 ± 0.16 [cd]	6.20 ± 0.18 [ef]	*6/10 M. humilis*<br>*4/10 S. cerevisiae*	*Kazachstania 100*
**SD103**	7.63 ± 0.01 [g]	6.83 ± 0.04 [c]	*10/10 S. cerevisiae*	*Saccharomyces 100*
**SD104**	8.56 ± 0.06 [c]	6.46 ± 0.09 [de]	*7/10 S. cerevisiae*<br>*3/10 M. unispora*	*Kazachstania 40; Pichia 35; Saccharomyces 25*
**SD105**	7.93 ± 0.10 [f]	5.56 ± 0.08 [h]	*7/10 S. cerevisiae*<br>*3/10 M. humilis*	*Kazachstania 100*
**SD106**	8.33 ± 0.06 [e]	7.20 ± 0.01 [b]	*10/10 T. delbrueckii*	*Pyrenophora 40; Papiliotrema 30; Saccharomyces 15; Cystofilobasidium 10; Tilletia 5*
**SD107**	7.66 ± 0.14 [g]	6.16 ± 0.01 [ef]	*10/10 S. cerevisiae*	*Kazachstania 100*
**SD108**	8.89 ± 0.02 [b]	5.59 ± 0.06 [gh]	*4/10 S. cerevisiae*<br>*6/10 P. fermentans*	*Wickerhamomyces 80; Saccharomyces 10; Cladosporium 5; Pyrenophora 5*
**SD109**	8.38 ± 0.07 [de]	6.29 ± 0.01 [ef]	*4/10 S. cerevisiae*<br>*6/10 P. fermentans*	*Saccharomyces 100*

LAB and yeast counts are expressed as mean ± standard deviation (Log CFU/g). Identified isolates are reported as the number of colonies assigned to each species out of 10 total isolates per sample (culture-based method). Relative abundances (%) of fungal genera were determined by ITS metabarcoding; values represent the percentage contribution of each genus to the total fungal community within each sample. Different lowercase superscript letters within each column indicate statistically significant differences among samples ($p < 0.05$) as determined by multiple comparison testing.

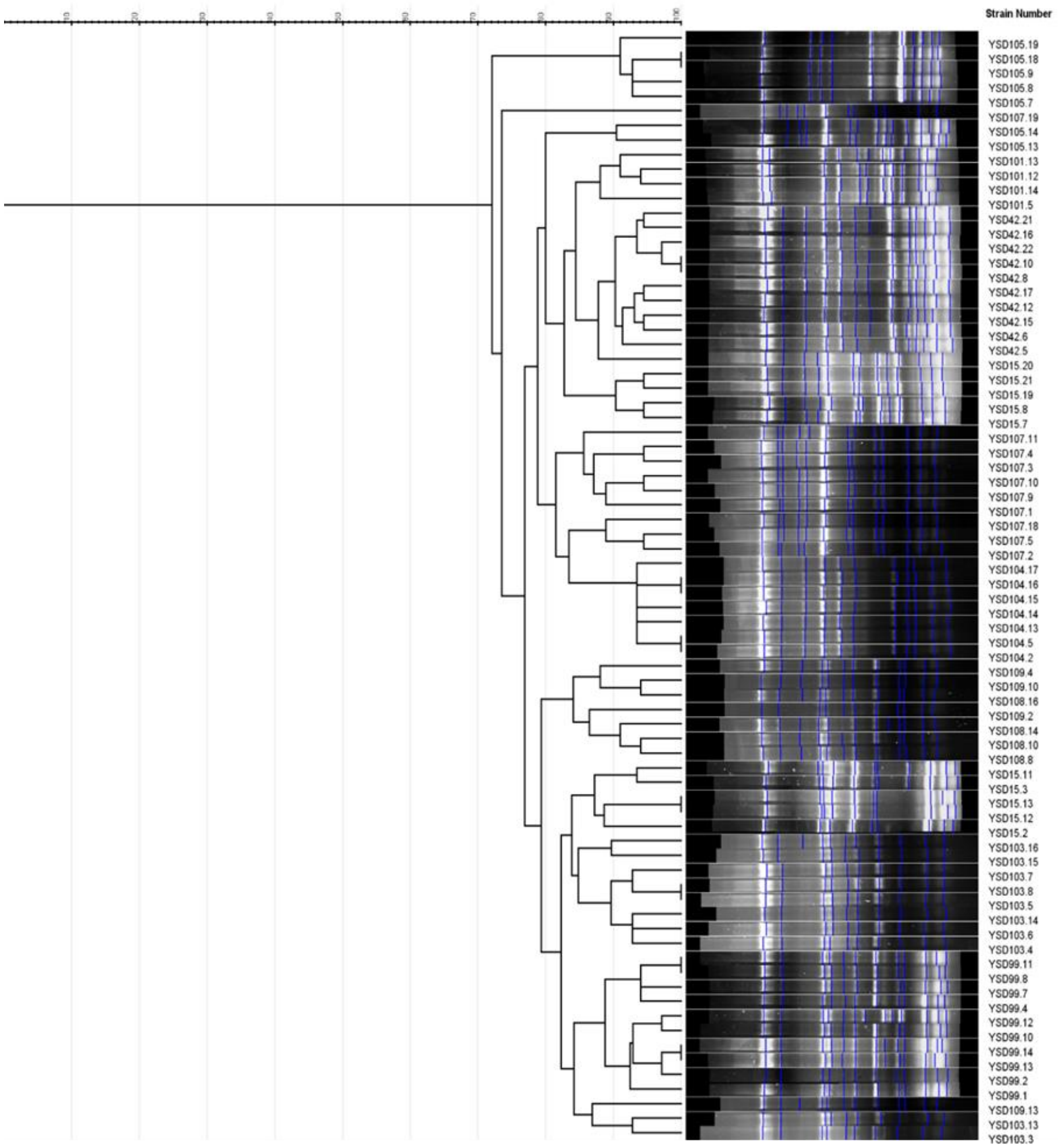

Figure S1. Dendrogram obtained from inter-delta PCR profiles of *Saccharomyces cerevisiae* isolates, generated using the Dice correlation coefficient with a tolerance of 1.5, showing the genetic relationships among the analysed strains.
